# Combining global genome and transcriptome approaches to identify the candidate genes of small-effect quantitative trait loci in collagen-induced arthritis

**DOI:** 10.1186/ar2108

**Published:** 2007-01-23

**Authors:** Xinhua Yu, Kristin Bauer, Dirk Koczan, Hans-Jürgen Thiesen, Saleh M Ibrahim

**Affiliations:** 1 Immunogenetics Group, University of Rostock, Schillingallee, 18055 Rostock, Germany; 2 Institute for Immunology, University of Rostock, Schillingallee, 18055 Rostock, Germany

## Abstract

Quantitative traits such as complex diseases are controlled by many small-effect genes that are difficult to identify. Here we present a novel strategy to identify the candidate genes for small-effect quantitative trait loci (QTL) in collagen induced arthritis (CIA) using global genome and transcriptome approaches. First, we performed genome linkage analysis in F2 progeny of the CIA susceptible and resistant strains to search for small-effect QTL. Second, we detected gene expression patterns of both strains during CIA. The candidate genes were identified using three criteria: they are located in a genomic region linked to CIA; they are disease-specific differentially expressed during CIA; and they are strain-specific differentially expressed regarding the two parental strains. Eight small-effect QTL controlling CIA severity were identified. Of 22,000 screened genes, 117 were both strain-specific and disease-specific differentially expressed during CIA. Of these 117 genes, 21 were located inside the support intervals of the 8 small-effect QTL and thus were considered as candidate genes.

## Introduction

Susceptibility to most complex diseases is controlled by many genes, each having a small effect on the disease. One example is rheumatoid arthritis (RA), a common complex multifactorial autoimmune disease. Several studies have been carried out to detect the genetic basis of RA, and more than 30 genomic regions have shown evidence of linkage to the disease. Most of these genomic regions did not reach a genome-wide significant threshold value of linkage, with *P *values between 0.05 and 0.001 [1-5]. Thus, these loci only have a small effect on RA. Small genetic contributions could also be seen from the susceptibility genes of RA identified so far, including *HLA-DR4*, *PADI4*, *PTPN22 *and *FCRL3 *[6-9]. Except for *HLA-DR4*, which is strongly associated with RA, all the other susceptibility genes have only a small effect on the disease. In the mouse model of RA, small genetic contributions are also often observed. For example, in a previous study, we carried out a genome screen to identify the quantitative trait loci (QTL) in collagen-induced arthritis (CIA), which is a widely used animal model of RA. Only one QTL, *Cia2*, was identified for the phenotype of CIA severity, but this QTL contributes to only 16% of the phenotype variations for CIA susceptibility in F2 progeny [10]. This suggests that there must be other susceptibility genes whose contributions were not big enough to reach the stringent significance threshold value of linkage analysis.

One aim of using animal models for complex diseases is to detect the genetic basis of these diseases. With controllable environmental factors as well as the known genetic background, animal models are powerful tools to search for susceptibility genes for complex diseases, and have been intensively employed for that purpose. More than 27,000 QTL have been identified in the mouse genome since the first QTL was identified at the beginning of the 1990s [11]. By 2005, approximately 20 quantitative trait genes (QTGs) in the mouse genome had been identified [12,13]. Interestingly, most QTGs identified in animal models have the causal polymorphisms in the protein-coding region [14], which provoke protein structure changes or protein deficiency. This suggests, on the one hand, that small-effect QTL are difficult to identify with traditional strategies and, on the other hand, that the polymorphisms regulating gene expression might only slightly affect the quantitative traits, and thus are more difficult to identify.

Microarray-based global gene expression is a powerful technique for investigating complex diseases. During disease development, genes involved in the disease are likely to be differentially regulated. Therefore, signature genes of the diseases could be identified by detecting the expression patterns of the disease-related cells/tissues and their ideal controls. In the past decade, many studies applied this technique to study both RA and its animal models [15-22]. Indeed, genes involved in arthritis show distinct expression patterns in certain tissues and pathological stages of the disease. Genes involved in immunoinflammatory responses were differentially expressed in the blood cells in RA patients [18]. Chemokines and adhesion molecules were upregulated in the joint at the initiation phase of arthritis in animal models [21,22], while genes involved in cartilage destruction and bone erosion were differentially expressed at the late phase of arthritis in animal models of RA [15,16]. Besides detecting genes involved in complex diseases, microarrays could also be used to detect the genetic polymorphisms regulating gene expression because differential expressions between two strains might be the result of a polymorphism located in regulatory elements.

To identify the small-effect QTL of CIA as well as the potential candidate genes inside them, we investigated CIA genetically susceptible and resistant strains at both the genome and transcriptome levels. At the genome level, F2 progeny of the CIA susceptible (DBA/1) and resistant (FVB/N) strains were generated and a genome-wide linkage analysis was performed to identify small-effect QTL. At the transcriptome level, we detected the gene expression patterns of both the DBA/1 and FVB/N strains at four different phases of CIA. The potential candidate genes were identified based on three criteria: they are located within the genomic region linked to CIA; they are disease-specific differentially expressed during CIA; and they are strain-specific differentially expressed between the two parental strains during CIA.

## Materials and methods

### Animals, immunisation and assessment of arthritis

Both DBA/1 and FVB/N mice used in this study were obtained from the Jackson Laboratory and kept in a climate-controlled environment with 12 hour light/dark cycles in the animal facility at the University of Rostock. All animal experiments were pre-approved by the State Animal Care Committee. CIA was induced in control and experimental animals according to established protocols described previously [10]. In brief, DBA/1J, FVB/N and (DBA/1J × FVB/N)F2 progeny were immunised at 8 to 12 weeks at the base of the tail with 125 μg of bovine Collagen II (Chondrex, Redmond, WA, USA) emulsified in CFA (DIFCO, Detroit, MI, USA). The clinical scoring of arthritis commenced 18 days after immunisation, and animals were monitored three times weekly for signs of CIA. Arthritis development was monitored in all four limbs using a three-score system per limb as described previously [10].

Eight-week old FVB/N and DBA/1J mice were used for the detection of gene expression. They were divided into four experimental groups according to the different phases of CIA, namely naive control (NC), post-immunisation (PI), onset of arthritis (OA) and chronic arthritis (CA) (Table 1). The NC group contained non-immunised mice that were sacrificed at the age of 8 weeks. The mice in the PI group were sacrificed on day 10 after immunisation. The mice in the OA group were sacrificed on day 35 after immunisation. Three FVB/N non-arthritic mice and three DBA/1 mice that showed signs of arthritis on day 33 or 34 after immunisation were sacrificed on day 35 after immunisation. The mice in the CA group were sacrificed on day 95 after immunisation. Three non-arthritic FVB/N mice and three DBA/1 mice that had developed arthritis for at least two months were sacrificed on day 95 after immunisation.

### Linkage analysis

Detailed information on genotyping of the genome screen has been described previously [10]. In short, we genotyped 290 F2 mice using 126 informative microsatellite markers covering the genome with an average inter-marker distance of 11.5 cM for 290 F2 progeny. All linkage analyses were performed with QTX Map manager software [23]. The main clinical phenotype of CIA, arthritis severity, was taken as phenotype. To detect the small-effect QTL, the threshold value of linkage was set as *P *= 0.05 (Chi-square test).

### Sample preparation and microarray hybridisation

Lymph nodes (LNs) draining the immunisation site were used for total RNA preparation. The total RNA was extracted from the tissue homogenates using a commercial kit in accordance with the provided protocol (QIGEN, Hilden, Germany). Analysis of gene expression was conducted using a U430A array (Affymetrix, Santa Clara, CA, USA) interrogating more than 22,000 genes. RNA probes were labelled in accordance with the manufacturer's instructions. Samples from individual mice were hybridised onto individual arrays. Hybridisation and washing of gene chips were done as previously described [16]. Fluorescent signals were collected by laser scan (Hewlett-Packard Gene Scanner).

### Microarray analysis

Normalisation of the expression level was done using Affymetrix software MAS 5, which is based on global scaling of total gene expression level per microarray. The normalised expression values were imported to and analysed by dCHIP [24]. Differentially expressed genes were identified by defining the appropriate filtering criteria in the dCHIP software as: lower 90% confidence boundary of fold change between the group means exceeded twofold; the absolute difference between the two groups exceeded 100; the *P *value threshold of the unpaired *t*-test was 0.05. The false discovery rate was established with a permutation test for each pairwise comparison to estimate the proportion of false-positive genes.

Hierarchical gene clustering was performed with dCHIP to characterise the gene expression patterns during CIA. The default clustering algorithm of genes was as follows: the distance between two genes is defined as 1 – r, where r is the Pearson correlation coefficient between the standardised expression values of the two genes across the samples used. To characterise the functional relationship between differentially expressed genes, Gene Ontology (GO) term classification incorporated in DNA-Chip Analyzer was performed. The significant level for a function cluster was set at *P *< 0.005, and the minimum size of a cluster was three genes.

## Results

### Small-effect QTL of CIA in (DBA/1 × FVB/N) F2 progeny

In a previous study, we carried out a genome screen to identify QTL controlling CIA susceptibility in (DBA/1 × FVB/N) F2 progeny. For the phenotype of arthritis severity, only one QTL, *Cia2*, was identified, with a highly significant logarithm of the odds (LOD) score of 12 [10]. However, *Cia2 *contributed to only 16% of the phenotype variations, indicating that there should be some small-effect QTL whose contributions to CIA were not big enough to reach the significant threshold value of linkage. To identify these potential small-effect QTL, we reanalyzed the data using a lower threshold value of linkage (*P *= 0.05). We reasoned that since the main candidate gene of *Cia2*, complement component C5 (*Hc*), was proven to be essential for CIA development and because the FVB/N strain is C5 deficient [10,25], some small-effect QTL might be masked by *Cia2*. To exclude the masking effect of C5, we performed linkage analysis with 3 datasets, the first containing all 290 F2 progeny, the second 77 C5^+/+ ^F2 progeny and the third 133 C5^+/- ^F2 progeny. Eight genomic regions were linked to the phenotype of CIA severity (loci 1 to 8, Table 2), with *P *values varying between 0.043 and 0.003. These eight small-effect QTL were located on chromosomes 5, 6, 7, 10, 11, 16, and 17. Five loci were identified in at least two datasets. Of the eight loci, five had DBA/1 as the susceptibility allele, and three had FVB/N as the susceptibility allele.

Lander and Botstein [26] suggested a LOD score of between 2 and 3 to ensure an overall false positive rate of 5%, which means that using a lower threshold value will prevent false negative QTL at the expense of increasing false positive QTL. Being aware of this, we examined these genomic regions to search whether they, or their syntenic genomic regions on the human genome, have been previously linked to arthritis. Four small-effect QTL overlapped with arthritis QTL on the mouse genome identified previously. Locus 1 and 2 overlap with *Cia13 *and *Cia14*, which control severity of CIA in (DBA/1 × BALB/C) F2 progeny [27]. Locus 5 located on chromosome 10 overlaps with *Cia8*, which was identified in (DBA/1 × B10.Q) F2 progeny [28]. Locus 6 overlaps with *Pgia7*, which controls susceptibility to proteoglycan-induced arthritis (PGIA) and was identified in (BALB/C × DBA/2) F2 progeny [29]. The syntenic genomic regions of five small-effect QTL on the human genome have been reported to be linked to RA. These are genomic regions 22q11 and 12p13-q24 on chromosome 22 and 12 (the counterparts of locus 2), 12p13-pter on chromosome 12 (the counterpart of locus 3), 21q22-qter and 10q22-23 on chromosome 21 and 10 (the counterparts of locus 5), 17q21-25 on chromosome 17 (the counterpart of locus 6) and 3q29-qter on chromosome 3 (the counterpart of locus 7) [2,4].

### Strain-specific differentially expressed genes

We detected the gene expression profiles using three mice per group, which is a small number. Being aware of the importance of data reproducibility, we determined the coefficient of variation (CV) to measure data variability. The CV for each gene on the chip and the mean CV for the entire probe set were calculated. The mean CV ranged between 18.4% and 25.8% for all experimental groups, and this relatively low CV indicated that these data could be used for further analysis (Table 1).

**Figure 1:**
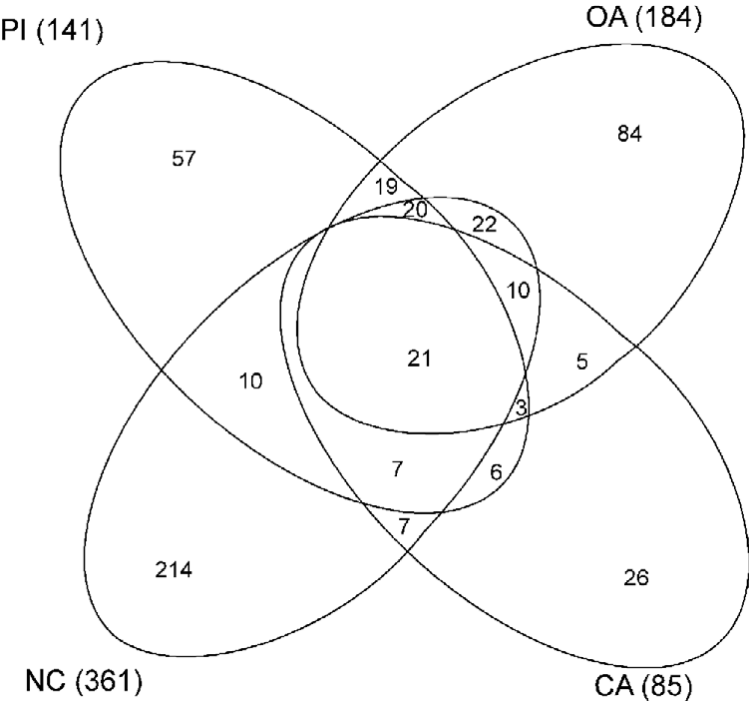
Differentially expressed genes between collagen-induced arthritis (CIA) susceptible and resistant strains. Comparison of the gene expression between the DBA/1 and FVB/N strains was performed at four phases of CIA. Of the 22,000 screened genes, 509 were differentially expressed between both strains at one or more phases of CIA, including 361 genes at the naive control (NC) phase, 141 genes at post-immunisation (PI) phase, 184 genes at the onset of arthritis (OA) phase and 85 genes at the chronic arthritis (CA) phase. The Venn diagram indicates the number of overlapping genes differentially expressed at different phases of CIA.

To search for strain-specific differentially expressed genes, we performed comparisons of gene expression between the DBA/1 and FVB/N strains at all four phases of CIA, including NC, PI, OA and CA. For the naive mice without immunisation, 361 genes were differentially expressed between the two strains. On day 10 after immunisation, when both strains did not show any sign of the disease, 141 genes were differentially expressed. After DBA/1 mice developed CIA, 184 and 85 differentially expressed genes were identified between these two strains at the onset and chronic phases, respectively. When the lists of the differentially expressed genes at the four phases were merged and overlapping genes were excluded, 509 genes were identified (Additional file 1). Twenty-one genes consistently showed differential expression between the two strains at all phases. Besides these 21 genes, only 3 additional genes were strain-specific differentially expressed during the 3 phases after CIA induction (PI, OA and CA; Figure 1).

### Disease-specific differentially expressed genes

**Figure 2:**
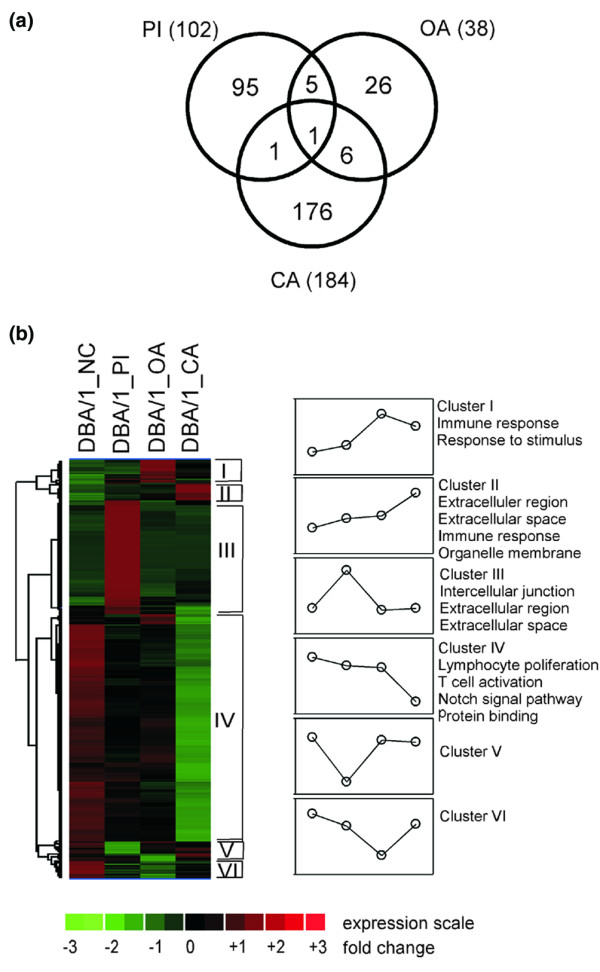
Differentially expressed genes in the DBA/1 strain during collagen-induced arthritis (CIA). Three experimental conditions, post-immunisation (PI), onset of arthritis (OA) and chronic arthritis (CA), were compared with naïve control (NC) to search for differentially expressed genes. **(a) **Venn diagram indicating the number of overlapping genes differentially expressed at three phases of CIA. **(b) **Hierarchical clustering of the 310 differentially expressed genes. The left panel shows the distribution of relative gene expression across the hierarchical tree structure. Rows represent individual genes; columns represent individual value of triplicate samples for each experimental group. Each cell in the matrix represents the expression level of a gene, with red and green indicating transcription levels above and below the normal values for that gene, respectively. Four sample groups are indicated above the expression matrix. Six basic gene clusters (clusters I to VI) were yielded by the analysis according to the gene expression pattern during CIA. The expression patterns of the gene cluster are graphed and the major biological activities for each cluster that were examined by functional clustering analysis are indicated on the right.

To identify the disease-specific differentially expressed genes in CIA, we detected the genes that were differentially expressed in LNs during CIA in the susceptible strain. Three experimental conditions, PI, OA and CA, were compared with the NC group. On day 10 after immunisation, 102 genes were differentially expressed – most of them were upregulated (78 out of 102) – while at the onset phase of the disease, only 26 genes were differentially expressed. At the chronic phase of the disease, 184 differentially expressed genes were identified, with 156 downregulated genes. Only one gene was differentially expressed at all three phases of CIA. Besides this gene, five, one and six differentially expressed genes were shared by PI with OA, PI with CA and OA with CA, respectively (Figure 2a). Taken together, 310 disease-specific differentially expressed genes were differentially regulated during CIA in DBA/1 mice (Additional file 2).

To further characterise the gene expression pattern during CIA, we performed hierarchical cluster analysis for these 310 genes. Six gene clusters were identified (clusters I to VI, Figure 2b), each with a distinct gene expression pattern during CIA. Cluster I contains 16 genes, representing genes that were upregulated after induction of CIA. The expression of these genes reached a peak at the onset phase of the disease and functional clustering results revealed that they are related to the immune response. Cluster II contains 12 genes whose expression was gradually upregulated and reached a peak at the chronic phase of CIA. These genes are mainly related to the immune response, organelle membrane and extracellular region and space. Cluster III contains 78 genes that were only upregulated at the PI phase. These genes are related to the intercellular junction. More than half of the genes (156 of 310) belong to cluster IV and represent genes specifically downregultaed at the chronic phase. These genes are functionally related to lymphocyte proliferation, T cell activation, protein binding as well as the notch signal pathway. Cluster V contains eight genes downregulated at the PI phase. Cluster VI contains 18 genes downregulated at the OA phase. The GO term classification showed no functional cluster that was significantly enriched in these two gene clusters.

### Candidate genes for the small-effect QTL of CIA

**Figure 3:**
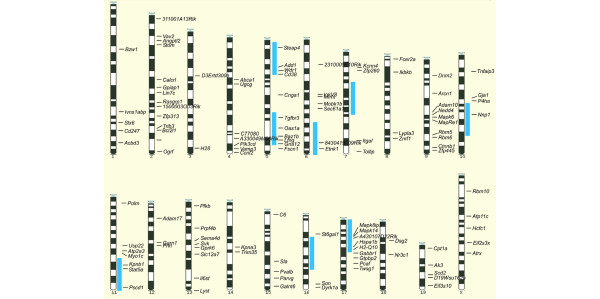
Visualisation of all the chromosomal locations of the small-effect QTL identified in this study (blue bar) as well as 120 genes of interest from gene expression profiling (black letters). The positions of the 120 genes and the peak markers of the QTL were retrieved from Ensembl [30]. Confidence intervals of all the QTL were set as 40 Mb.

To identify candidate susceptibility genes for the CIA small-effect QTL, we compared the list of strain-specific differentially expressed genes with the list of disease-specific differentially expressed genes; 117 genes were shared by both lists (Additional file 3). Figure 3 visualises positions of the 117 genes retrieved from Ensembl [30] in relation to the 8 small-effect QTL. The eight loci were located on 7 chromosomes, 5, 6, 7, 10, 11, 16 and 17. Since the confidence intervals of QTL in F2 progeny are around 20 cM [26], we used 40 Mb as the confidence intervals for all loci. Twenty-one genes were located in the confidence intervals of six of the eight QTL. We located 5, 4, 2, 1, 3 and 6 potential candidate genes within the confidence intervals of loci 1, 2, 3, 5, 6 and 8, respectively, while no candidate gene was identified for loci 4 and 7. Table 3 summarises the 21 candidate genes identified in this study. Two genes, *hspa1a *and *Oas1a*, were upregulated at the OA phase of CIA and *Oas1a *was also upregulated at the PI phase. Except for these two genes, all other 19 genes were downregulated at the chronic phase of CIA. All genes, except *hspa1a*, showed expression differences between the two strains at the NC phase. Five genes were differentially expressed at all phases of CIA, including *H2-Q10*, *Mapk14*, *Pscd1*, *Kpnb1 *and *Wdr1*. Among these five genes, *H2-Q10 *had a consistently higher expression in the DBA/1 than the FVB/N strain in all CIA phases, while the other four genes had a higher expression in the DBA/1 strain at the early stages, including NC, PI and OA, but a lower expression at the chronic phase. GO term classification analysis revealed that the functional cluster of protein kinase cascade was significantly enriched in the 21 candidate genes. This functional cluster contained four genes, including *Mapk14*, *Mapk8ip3*, *Stat5a *and *Gna12*.

## Discussion

In this study, we attempted for the first time to identify small-effect QTL in an F2 progeny. Small-effect QTL are defined as those reaching the threshold value of *P *= 0.05 but that did not reach the significant threshold value suggested by Lander and Botstein [26]. Although not significant, there is evidence that most of the eight small-effect QTL likely contain susceptibility genes for CIA. First, we performed the linkage analysis in three datasets, including all 290 F progeny, 76 C5+/+ F2 progeny and 133 C5+/- F2 progeny. Five of the eight small-effect QTL were identified in at least two datasets, suggesting that these QTL are reproducible. Second, many QTL identified in the present study overlap with arthritis QTL previously identified, including loci 1, 2, 5 and 6. In addition, syntenic analysis revealed that the counterpart genomic regions on the human genome of many of these eight QTL are linked to RA.

For five of the eight small-effect QTL the DBA/1 alleles are the arthritis-enhancing alleles, while the FVBN alleles are the arthritis-enhancing alleles in the other three QTL, indicating that some susceptibility genes could come from the resistant strain. Interestingly, loci 2 and 7 partially overlap with two arthritis-related QTL identified by us in the same F2 progeny [10]. Locus 2 was located at the same genomic region as *Cia27*, a QTL controlling IgG2a antibody levels to collagen II. Recently, we have refined this QTL into a 4.1 Mb genomic region and showed that a gene within this region regulates CIA severity by controlling the IgG2a antibody levels to collagen II [31]. Locus 7 on chromosome 16 overlaps with *Lp1*, which controls lymphocyte proliferation. Furthermore, according to our unpublished data, loci 8 on chromosome 17 controls lymphocyte adherence during development of CIA. Therefore, the gene within the small-effect QTL could affect CIA severity through controlling arthritis-related phenotypes.

Several studies have been carried out to detect gene expression during CIA, all of which used joints as the target tissue [15,16,21,22]. This study, for the first time, detected gene expression in LNs during CIA. We present an extensive study of gene expression patterns in LNs of both genetically susceptible and resistant strains at four different phases of CIA. In both strains, differentially regulated genes were highly concentrated at the PI and CA phases, and only a small number of genes were differentially expressed in two or three phases. This indicates that biological responses in LNs were stronger in the PI and CA phases than in the OA phase, and the responses at different phases were different. When comparing the susceptible to the resistant strain, the biggest difference was found in one cluster of genes (cluster IV, Figure 2). These genes had a higher expression in DBA/1 than in FVB/N at the early phases of CIA (NC, PI and OA) and the opposite expression pattern in the CA phase. GO term classification revealed that these genes were related to lymphocyte proliferation and activation, suggesting that lymphocytes in the DBA/1 strain are more activated than those in the FVB/N strain. However, this difference is not CIA specific because the expression difference between the two strains existed in mice without immunisation. Additionally, some genes related to the immune response were upregulated in the DBA/1 strain but not in the FVB/N strain during CIA. These differences could explain why a higher antibody response to collagen II occurred in the DBA/1 strain compared to the FVB/N strain, and might partially explain the difference of the susceptibility to CIA between both strains.

Twenty-one genes were identified as potential candidate genes for six of the eight small-effect QTL according to their gene expression patterns during CIA and their genomic locations. No candidate genes were located in QTL 4 and 7, suggesting that QTG polymorphisms of the susceptibility genes inside these two QTL might not affect the phenotype by regulating gene expression. Two of the 21 candidate genes were reported to be involved in arthritis. *Mapk14*, a candidate gene for locus 8 and also called p38 mitogen-activated protein kinase (MAPK) alpha, regulates the production of arthritis-essential cytokines, such as tumour necrosis factor and interleukin-1 [32]. Moreover, inhibitors of p38 MAPK could attenuate CIA in rats [33], and p38 MAPK is becoming a potential therapeutic target in RA [32]. *Stat5a*, a candidate gene for loci 6, is an essential molecule for lymphoid development and differentiation [34]. *Stat5a*-deficient mice were reported to lose tolerance, resulting in the development of autoimmune diseases. *Stat5a *is suggested to contribute to tolerance through maintenance of the CD4+CD25+ regulatory T cell population [35].

Therefore, we have presented a strategy to identify small-effect QTL and search for potential candidate genes within them. However, it is noteworthy that the low statistical threshold and small number of animals per group could lead to some false positive results. On the genome level, some of the eight small-effect QTL identified using a very low threshold value (*P *< 0.05) could be false positives. For example, locus 4 was identified with a low *P *value and does not overlap with any previously identified arthritis QTL. On the transcriptome level, the small number of animals per group and the low threshold used to detect gene expression could also result in false positives in the differentially expressed genes. Furthermore, the differential expression of a gene could result not only from allele difference between two strains, but also from other factors. Therefore, our findings should be confirmed in future studies.

## Conclusion

We present a strategy to search candidate genes for small-effect QTL. With this strategy, we identified 21 candidate genes for 8 small-effect QTL regulating CIA susceptibility. Our future studies will be carried out using two approaches. The first is generating congenic animals for promising small-effect QTL that have relatively high *P *values and overlap with previously-identified arthritis QTL. The second approach is investigating candidate genes using both mouse and human studies. Candidate genes will be selected according to their function and polymorphism between the two strains. Thereafter, we will generate knock-out mice to investigate the role of the genes in CIA. For the loci whose counterparts on the human genome are linked to RA, we will investigate the candidate genes using case-control association studies in RA cohorts.

## Abbreviations

CA = chronic arthritis; CIA = collagen-induced arthritis; CV = coefficiant of variation; GO = Gene Ontology; LN = lymph node; LOD = logarithm of the odds; MAPK = mitogen-activated protein kinase; NC = naive control; OA = onset of arthritis; PI = post-immunisation; QTG = quantitative trait gene; QTL = quantitative trait loci; RA = rheumatoid arthritis.

## Competing interests

The authors declare that they have no competing interests.

## Authors' contributions

XY and KB performed the animal experiments. DK and HJT performed gene expression profiling experiments. XY and DK performed the bioinformatic analysis. SI conceived and designed the experiment. XY and SI drafted the manuscript. All authors read and approved the final manuscript.

## Supplementary Material

Additional file 1 Summary information on the 509 genes differentially expressed between DBA/1 and FVB/N strains. Comparison was performed between two strains at four stages of CIA, NA, PI, OA and CA. Three criteria were applied for selecting the differentially expressed genes: the lower 90% confidence bound of fold change between the group means exceeded twofold; the absolute difference between the two groups exceeded 100; the *P *value threshold of the unpaired *t*-test was 0.05 Click here for file

Additional file 2 Summary information on the 311 genes differentially expressed in joints in the DBA/1 strain during CIA. Pairwise comparisons were performed by comparing PI, OA and CA with NA. Three criteria were applied for selecting the differentially expressed genes: the lower 90% confidence bound of fold change between the group means exceeded twofold; the absolute difference between the two groups exceeded 100; the *P *value threshold of the unpaired *t*-test was 0.05 Click here for file

Additional file 3 Summary information on the 117 genes that were strain-specific differentially expressed and were dysregulated in the DBA/1 strain during CIA. The physical positions of the genes were retrieved from Ensembl [30] Click here for file

## Figures and Tables

**Table 1:** Experimental groups used for gene expression profiling

Group	Treatment	RNA source	No. of mice	CV (percentage)
DBA/1_NC	None	LN from naive control DBA/1	3	22.8
DBA/1_PI	CFA + collagen II	LN from DBA/1 at day 10 after immunisation	3	20.4
DBA/1_OA	CFA + collagen II	LN from DBA/1 mice at day 35 after immunisation	3	21.7
DBA/1_CA	CFA + collagen II	LN from DBA/1 mice at day 95 after immunisation	3	25.8
FVB/N_NC	None	LN from naive control FVB/N	3	21.1
FVB/N_PI	CFA + collagen II	LN from FVB/N at day 10 after immunisation	3	22.6
FVB/N_OA	CFA + collagen II	LN from FVB/N mice at day 35 after immunisation	3	18.4
FVB/N_CA	CFA + collagen II	LN from FVB/N mice at day 95 after immunisation	3	21.1

CA, chronic arthritis; CFA; complete Freund's adjuvant; CV, coefficient of variation; LN, lymph node; NC, naive control; OA, onset of arthritis; PI, post-immunisation.

**Table 2:** Summary of the small-effect QTL identified in this study

Loci	Chromosome	Peak marker	Position (Mb)	*P *value	Susceptibility allele	Overlapping QTL in mouse models of RA	Snyternic region linked to RA (linked marker)
1^a,b,c^	5	D5Mit348	23,9	0.00691	DBA/1	Cia13	
2^b^	5	D5Mit425	119,1	0.04397	DBA/1	*Cia14*	*22q11 *(*D22S264*), *12p13-q24 *(*D12S95*)
3^a,b,c^	6	D6Mit14	145,7	0.00315	FVB/N		*12p13-pter *(*D12S99*)
4^a,b,c^	7	D7Mit248	59,3	0.03250	DBA/1		
5^a,c^	10	D10Mit261	85,1	0.00647	DBA/1	*Cia8*	*21q22-qter *(*D21S268*), *10q22-23 *(*D10S2327*)
6^b,c^	11	D11Mit214	115,2	0.00521	DBA/1	*Pgia7*	*17q21-q25 *(*D17S1301*)
7^c^	16	D16Mit138	45,4	0.01499	FVB/N		*3q29-qter *(*D3S1311*)
8^c^	17	D17Mit197	18,3	0.02994	FVB/N		

^a^Identified in all F2 290 progeny. ^b^Identified in 76 C5+/+ F2 progeny. ^c^Identified in 133 C5+/- F2 progeny. QTL, quantitative trait loci; RA, rheumatoid arthritis.

**Table 3:** Summary of the small-effect QTL candidate genes

Gene	Chr.	Position (Mb)	Description	Difference between DBA/1 and FVB/N strains		Difference during CIA in DBA/1 strain	
				Phase	Fold change^a^	Phase	Fold change^b^

Steap4	5	7.9	STEAP family member 4	NC/PI/CA	12.9/7.1/-5.9	CA	-8.3
*Add1*	5	33.6	Adducin 1 (alpha)	NC/PI	5.1/3.3	CA	-4.6
*Wdr1*	5	37.8	WD repeat domain 1	NC/PI/OA/CA	4.9/3.5/2.4/-3.2	CA	-7.1
*Cd38*	5	43.1	CD38 antigen	NC/PI	5.7/4.0	CA	-3.6
*Tgfbr3*	5	106.2	Transforming growth factor, beta receptor III	NC/PI/CA	8.7/6.9/-3.2	CA	-3.9
*Oas1a*	5	120.1	2'-5' Oligoadenylate synthetase 1A	NC	-2.5	OA	2.7
*Baz1b*	5	134.1	Bromodomain adjacent to zinc finger domain, 1B	NC/PI	5.9/5.7	CA	-6.7
*Lfng*	5	139.5	Lunatic fringe gene	NC/PI/OA	8.8/4.6/2.5	CA	-7.7
*Gna12*	5	139.7	Guanine nucleotide binding protein, alpha 12	NC/PI/OA	4.1/3.7/3.3	CA	-3.5
*8430419L09Rik*	6	135.2	RIKEN cDNA 8430419L09 gene	NC/PI/OA	17.8/12.8/3.3	CA	-4.2
*Etnk1*	6	143.2	Ethanolamine kinase 1	NC/PI/OA	4.6/3.9/2.6	CA	-5.6
*Nnp1*	10	78.05	Novel nuclear protein 1	NC	3.0	CA	-3.5
*Kpnb1*	11	97.2	Karyopherin (importin) beta 1	NC/PI/OA/CA	12.1/7.3/3.2/-20	CA	-50
*Stat5a*	11	100.9	Signal transducer and activator of transcription 5A	NC/PI	5.3/3.0	CA	-4.6
*Pscd1*	11	118.2	Pleckstrin homology, Sec7 and coiled-coil domains 1	NC/PI/OA/CA	4.7/3.6/2.4/-4	CA	-11
*Mapk8ip3*	17	23.07	Mitogen-activated protein kinase 8 interacting protein 3	NC/PI	3.9/2.5	CA	-3.1
*Mapk14*	17	26.8	Mitogen activated protein kinase 14	NC/PI/OA/CA	9.1/4.7/3.1/-14	CA	-33.3
*A430107D22Rik*	17	30.5	RIKEN cDNA A430107D22 gene	NC	3.7	CA	-5
*Hspa1a*	17	33	Heat shock protein 1A	PI/CA	-3.8/-4.6	PI/OA	3.8/5.4
*H2-Q10*	17	33.5	Histocompatibility 2, Q region locus 10	NC/PI/OA/CA	3.3/4.2/2.5/5.3	CA	-3
*Gabbr1*	17	35.1	Gamma-aminobutyric acid receptor 1	NC/PI	4.9/2.5	CA	-6.3

^a^Fold change calculated by comparing DBA/1 with FVB/N. ^b^Fold change calculated by comparing post-immunisation (PI), onset of arthritis (OA) and chronic arthritis (CA) with naive control (NC), respectively. Chr., chromosome; CIA, collagen-induced arthritis.
